# Sex differences in the frailty phenotype and mortality in the I-Lan longitudinal aging study cohort

**DOI:** 10.1186/s12877-024-04785-w

**Published:** 2024-02-23

**Authors:** Ya-Wen Lu, Chun-Chin Chang, Ruey-Hsing Chou, Wei-Ju Lee, Liang-Kung Chen, Po-Hsun Huang, Shing-Jong Lin

**Affiliations:** 1 Division of Interventional Cardiology, Cardiovascular Center, Taichung Veterans General Hospital, Taichung, Taiwan; 2 Division of Cardiology, Department of Medicine, Taipei Veterans General Hospital, Taipei, Taiwan; 3 Department of Critical Care Medicine, Taipei Veterans General Hospital, Taipei, Taiwan; 4 Center for Geriatrics and Gerontology, Taipei Veterans General Hospital, Taipei, Taiwan; 5 Department of Family Medicine, Taipei Veterans General Hospital Yuanshan Branch, Yi-Lan, Taiwan; 6 Cardiovascular Research Center, National Yang Ming Chiao Tung University, Taipei, Taiwan; 7 Institute of Clinical Medicine, National Yang Ming Chiao Tung University, Taipei, Taiwan; 8 Center for Healthy Longevity and Aging Sciences, National Yang Ming Chiao Tung University, Taipei, Taiwan; 9 Department of Geriatric Medicine, National Yang Ming Chiao Tung University, School of medicine, Taipei, Taiwan; 10 Taipei Heart Institute, Taipei Medical University, Taipei, Taiwan; 11 Division of Cardiology, Heart Center, Cheng-Hsin General Hospital, Taipei, Taiwan; 12 Taipei Municipal Gan-Dau Hospital, Taipei, Taiwan

**Keywords:** Frailty, Sex difference, Fried frailty index

## Abstract

**Background:**

Frailty is a common geriatric syndrome related to multiple adverse outcomes. Sex differences in its prevalence and impact on mortality remain incompletely understood.

**Methods:**

This study was conducted with data from the I-Lan Longitudinal Aging Study, in which community-dwelling subjects aged > 50 years without coronary artery disease or diabetes were enrolled. Sex disparities in phenotypically defined frailty and sex–morality predictor interactions were evaluated. Sex- and frailty-stratified analyses of mortality were performed.

**Results:**

The sample comprised 1371 subjects (51.4% women, median age 61 years). The median follow-up period was 6.3 (interquartile range, 5.8–7.0) years. The frailty prevalence did not differ between men (5.3%) and women (5.8%). Frail individuals were older and less educated and had poorer renal function than did non-frail individuals. Body composition trends differed between sexes, regardless of frailty. Relative to non-frail men, frail men had significantly lower body mass indices (BMIs; 24.5 vs. 23.4 kg/m^2^, *p* = 0.04) and relative appendicular skeletal muscle masses (7.87 vs. 7.05 kg/m^2^, *p* < 0.001). Frail women had significantly higher BMIs (25.2 vs. 23.9 kg/m^2^, *p* = 0.02) and waist circumferences (88 vs. 80 cm, *p* < 0.001) than did non-frail women. Frailty was an independent mortality predictor for men only [hazard ratio (95% confidence interval) = 3.395 (1.809–6.371), *p*_sex–frailty interaction_ = 0.03].

**Conclusion:**

Frailty reflected poorer health in men than in women in the present cohort. This study revealed sex disparities in the impact of frailty on mortality among relatively healthy community-dwelling older adults.

**Supplementary Information:**

The online version contains supplementary material available at 10.1186/s12877-024-04785-w.

## Introduction

Frailty is a complex geriatric syndrome representing cumulative physiological decline, increased vulnerability, and the depletion of health reserves [1–3]. The commonly used phenotypic Fried frailty index (FFI) consists of five criteria: unintentional weight loss, exhaustion, low energy expenditure, slow gait speed, and weak grip strength [4]. Frailty affects older adults and those in the middle years by FFI or deficits accumulation approach [5–7]. Physical frailty is related to unfavorable health-related outcomes in nearly 500,000 participants with a mean age of 56 and is probably associated with altering brain structure [8]. Epidemiological studies have demonstrated that frailty, although inconclusively defined, frailty is significantly associated with mortality, including middle-aged individuals with or without comorbidities [1, 9, 10].

Reported effects of sex on the prevalence of frailty and its association with mortality are inconsistent and highly variable due to the use of different frailty assessments and the diversity of study populations [1, 10]. In community-dwelling and institutionalized populations, frailty has been reported to be more prevalent among women than among men of the same age [11, 12]. In contrast, no sex difference in the prevalence of frailty was observed in the Korean population without diabetes [13]. Few studies have directly compared the frailty–mortality relationship according to sex [14–16]. A meta-analysis revealed that females live longer than males in the general population despite comorbidities or frailty. Still, the authors noted that the included studies exhibited high heterogeneity [11]. In contrast, another study showed no significant sex difference in all-cause mortality for frail older men and women relative to robust individuals [17]. The impact of frailty–sex interaction on mortality remains uncertain [18, 19].

Although the FFI criteria are used most in clinical practice and could identify the shirking syndrome [4], body weight and body composition in frail populations were contradictory, and few studies examined only in elderly populations [20–22]. Higher body fat and body mass index (BMI) are positively correlated with frailty in a total of 29,937 participants aged ≥50 years from 2 large cohorts [23]; however, there were potential sex differences in the defining of frailty and body composition characteristics. We performed this study to 1) differentiate biological factors of frailty between sexes and 2) directly compare the relationship between phenotypic frailty and all-cause mortality between relatively healthy middle-aged to older men and women in Taiwan.

## Methods

### Study population

The present retrospective cohort study was performed with data from the I-Lan Longitudinal Aging Study (ILAS), conducted with a cohort of community-dwelling adults aged > 50 years who were recruited randomly via household registration records in I-Lan County, Taiwan. The exclusion criteria were: (i) inability to cooperate or communicate with the investigators; (ii) refusal of consent; (iii) current institutionalisation; (iv) known active disease, such as cancer, sepsis, heart failure or chronic obstructive pulmonary disease, or functional dependence; (v) life expectancy < 6 months; and (vi) plan to leave I-Lan County in the near future. The participants were randomly sampled through the county’s household registrations in Youanshan Township of I-lan County. The selected residents were invited to participate by mail or telephone invitations extended by the research team. From August 2011 to August 2013, well-trained research nurses interviewed all potential participants in person to assess their eligibility before they provided written informed consent. The ILAS design, participant recruitment and data collection have been reported elsewhere [24]. The study was conducted according to the Declaration of Helsinki and was approved by the institutional review board of National Yang-Ming Chiao Tung University (no. YM103008).

### Collection of anthropometric, demographic and laboratory data

A research nurse collected demographic and medical data (e.g. educational level, smoking habit, medical history) on the participants via personal interviews and medical records review. Participants’ brachial blood pressure was measured with a mercury sphygmomanometer after they had rested for at least 15 min. Peripheral blood samples were collected between 7 and 9 am after a ≥ 10-h fast for determination of the of haemoglobin A1c (HbA1c), fasting blood glucose (FBG), total cholesterol, high-density lipoprotein (HDL), low-density lipoprotein, triglyceride (TG) and uric acid (UA) concentrations using an automatic analyser (ADVIA 1800; Siemens, Malvern, PA, USA).

Metabolic syndrome (MS) was defined according to the criteria proposed by Taiwan’s Ministry of Health and Welfare as the presence of more than three of the following risk determinants: (i) waist circumference (WC) > 90 cm for men or > 80 cm for women; (ii) systolic blood pressure ≥ 130 mmHg, diastolic blood pressure ≥ 85 mmHg or antihypertensive agent use; (iii) HDL concentration < 40 mg/dL for men or < 50 mg/dL for women; (iv) TG concentration ≥ 150 mg/dL; and (v) FBG level ≥ 100 mg/dL or antihyperglycemic agent use. Central obesity was evaluated with the WC and waist-to-height ratio (WHtR) [25]. Chronic kidney disease was defined as estimated glomerular filtration rate (eGFR) < 60 mL/min/1.73 m^2^, calculated using the Chronic Kidney Disease Epidemiology Collaboration equation [26]. Hypertension was defined as self-reported current antihypertensive medication prescription, systolic blood pressure ≥ 140 mmHg or diastolic blood pressure ≥ 90 mmHg. Multimorbidity was identified using the Charlson comorbidity index (CCI) [27].

### Assessment of body composition, muscle strength and physical performance

Participants’ total fat mass and fat-free lean body mass were measured by whole-body dual-energy X-ray absorptiometry using a Lunar Prodigy device (GE Healthcare, Madison, WI, USA). The total body fat percentage was calculated as the total fat mass divided by the total body mass multiplied by 100 [28]. Handgrip strength was measured with a digital dynamometer (Smedlay’s Dynamo Meter; TTM, Tokyo, Japan), with the best performance among three trials recorded [29]. The appendicular skeletal muscle mass (ASM) was calculated as the sum of the four limbs’ lean soft-tissue mass, determined by dual-energy X-ray absorptiometry. The height-adjusted muscle index, or relative appendicular skeletal muscle mass (RASM), was calculated by dividing the ASM by the height squared [28]. Low muscle mass was defined according to the recommendation of the Asian Working Group for Sarcopenia [30] as RASM < 7.0 kg/m^2^ for men and < 5.4 kg/m^2^ for women.

### Physical frailty assessment

Physical frailty was identified using the FFI criteria [4], and participants’ physical activity was assessed using the Taiwanese version of the short-form Instrumental Physical Activity Questionnaire (IPAQ) [31]. The interviewer measured participants’ gait speed using the 6-m straight-line walking test and a stopwatch (HS-70 W; Casio Computer Co., Ltd., Tokyo, Japan) [32]. The low activity level, weakness and slowness parameters were defined by the lowest quintiles of the IPAQ score, sex-specific handgrip strength and sex-specific gait speed, respectively. Unintentional weight loss was ascertained using the threshold of ≥4.5 kg in the past year. Exhaustion was defined by self-reported ease of becoming weary or tired or inability to carry out ordinary daily tasks within the past week. Participants who fulfilled at least three FFI criteria were classified as frail, those who fulfilled one or two criteria were classified as prefrail and those who fulfilled no criterion were classified as robust. Non-frailty was defined as robustness and prefrailty.

### Determination of mortality

Follow-up were conducted between January 2018 and December 2019 according to telephone calls, and participants’ vital status was determined by telephone interviews. As follow-up medical records were not available, the causes of mortality were unknown.

### Statistical analysis

Categorical variables are expressed as frequencies and percentages, non-normally distributed continuous variables are expressed as medians with interquartile ranges and normally distributed continuous variables are expressed as means ± standard deviations. The Mann–Whitney *U* test and Fisher’s exact test were used to compare continuous and categorical variables, respectively, between groups. Stepwise backward logistic regression was used to identify significant predictors of physical frailty. Cox proportional-hazard regression analysis was used to examine the effect of frailty status on mortality separately among men and women. Factors that were significant in univariate regression analyses (*p* < 0.2) were entered into a multivariable regression analysis. Hazard ratios (HRs) with 95% confidence intervals (CIs) for mortality risk were calculated. Sex- and frailty-stratified mortality comparisons were performed using the log-rank test and Kaplan–Meier method. We performed the interaction testing to quantify the influence of sex on the relationship between characteristics and physical frailty and mortality. We were missing lean body mass and total body fat percentage data on 3 participants in frail men and women, repspectively and 9 in nonfrail women, 26 in nonfrail men. There were no significant differences in sex, age, or physical frailty status between those with and without the DXA scan data. The statistical analyses were performed using the SPSS software (version 23.0; IBM Corporation, Armonk, NY, USA). Two-tailed *p* values < 0.05 were regarded as significant.

## Results

**Fig. 1:**
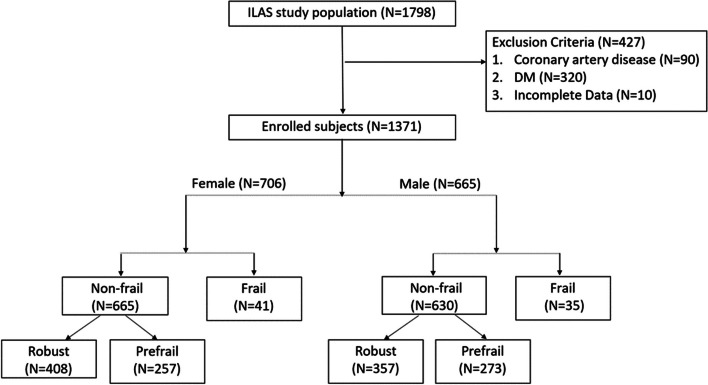
Flow chart of enrolled subjects

Of 1798 community-dwelling older adults attending initial interviews, 427 subjects were excluded from the ILAS because they had coronary artery disease (*n* = 90), diabetes (*n* = 320) or incomplete data (*n* = 10; Fig. 1). The study sample thus comprised 1371 participants (51.4% women) with a median age of 61.0 years. The median follow-up period was 6.3 (interquartile range, 5.8–7.0) years and 147 (10.7%) participants died. The subjects’ frailty prevalence was 5.5%, and pre-fail was 38.7%. Women were significantly younger and had fewer years of education, a lesser smoking prevalence, a lesser RASM, and lower blood TG and UA levels than men. Women had significantly higher total body fat percentage, HDL and HbA1c concentrations and eGFRs than men. The prevalence of frailty, mortality rate, hypertension and MS and the CCI value did not differ between the sexes (Table 1).

**Table 1:** Total patients separated by gender (*n* = 1371)

	Total *n* = 1371	Female *n* = 706	Male *n* = 665
Age (years)	61 (55.3–70.1)	60 (55–68.4)	62.7 (55.7–72.0)
Smoking (%)	251 (18.3)	24 (3.4)	227 (34.1)
Academic year	6 (2–9)	6 (0–9)	6 (5.5–12)
**Body Composition**			
BMI	24.2 (22.1–26.5)	23.9 (21.9–26.4)	24.4 (22.4–26.5)
Waist circumference (cm)	83 (77–90)	80 (74–86.6)	86 (81–92)
WHtR	0.52 (0.49–0.56)	0.52 (0.48–0.57)	0.52 (0.49–0.56)
Total body fat (%)_DXA	31.7 (24.9–37.8)	37.2 (33.0–41.3)	25 (20.5–29.1)
LBM (kg)	39.93 (34.25–47.55)	34.79 (32.58–37.24)	47.73 (43.69–51.96)
RASM (kg/m^2^)	6.87 (6.04–7.83)	6.17 (5.76–6.65)	7.84 (7.26–8.39)
Low muscle mass	146 (10.6)	87 (12.3)	59 (8.9)
**Underlying disease**			
Metabolic syndrome (%)	334 (24.4)	187 (26.5)	147 (22.1)
Hypertension (%)	465 (33.9)	230 (32.6)	235 (35.3)
CCI	0 (0–2)	0 (0–2)	0 (0–1.5)
**Medication**			
Anti-hypertension agents (%)	234 (17.1)	112 (15.9)	122 (18.3)
Statin (%)	59 (4.3)	38 (5.4)	21 (3.2)
**Laboratory data**			
Total Cholesterol (mg/dl)	198 (175–219)	204 (181–225)	191 (170–213)
HDL (mg/dl)	54 (46–63)	58 (50–69)	49 (43–57)
LDL (mg/dl)	120 (100–141)	121 (101–142)	118 (99–139)
Fasting glucose (mg/dl)	94 (88–100)	93 (88–99)	94 (89–100)
HbA1c (%)	5.7 (5.5–6.0)	5.8 (5.6–6.0)	5.7 (5.5–5.9)
Triglyceride (mg/dl)	101 (75–140)	97 (71–132.3)	107 (77–150)
UA	5.7 (4.8–6.7)	5.1 (4.4–5.9)	6.4 (5.5–7.4)
eGFR (ml/min/1.73m^2^)	91.6 (80.2–98.6)	94.21 (84.64–100.37)	88.03 (76.26–95.31)
**Non-frail**			
Robust	765 (55.8)	408 (57.8)	357 (53.7)
Pre-frail	530 (38.7)	257 (36.4)	273 (41.1)
**Frail**	76 (5.5)	41 (5.8)	35 (5.3)
**Mortality**	147 (10.7)	70 (9.9)	77 (11.6)

Values are median (25th–75th percentile) or *n* (%)

*BMI* body mass index: *WHtR* waist-to-height ratio: *BMD* bone mineral density: *LBM* lean body mass: *RASM* relative appendicular skeletal muscle: *CCI* Charlson’s Comorbidity Index: *HDL* high density lipoprotein: *LDL* low density lipoprotein: *HbA1c* hemoglobin A1c: *UA* uric acid: *eGFR* estimated glomerular filtration rate

Sex disparities stratified by frailty status are shown in Table 2. Frail subjects of both sexes were significantly older and had less education, poorer renal function, higher CCI values and higher mortality than did their non-frail counterparts. Among women, frail subjects had significantly lower total cholesterol and HDL levels and higher TG levels than did non-frail subjects. Frail men had lower HDL levels than did non-frail men, with no difference in any other lipid parameter or the FBG, HbA1c or UA level. Different trends in body composition were observed in men and women. Compared with non-frail women, frail women had significantly higher body mass indices (frail vs non-frail, 25.2 vs. 23.9 kg/m^2^, *p* = 0.02), WCs (frail vs non-frail, 88 vs. 80 cm, *p* < 0.001), and WHtRs (frail vs non-frail, 0.58 vs. 0.52, *p* < 0.001). In contrast, frail males had significantly lower BMIs (frail vs non-frail, 23.4 vs. 24.5 kg/m^2^, *p* = 0.04) and RASMs (frail vs non-frail, 7.05 vs. 7.99 kg/m^2^, *p* < 0.001) than did non-frail males, with no difference in the WHtR (0.54 and 0.51, respectively) or WC (87 and 85 cm, respectively). Significant BMI, WC, and WHtR interaction between sexes was detected. No sex disparity in low muscle mass was observed. The impact of frailness on mortality in men is more dominant than in women (women frail vs. non-frail 22.0 vs 9.2; men frail vs non-frail 48.6 vs 9.5).

**Table 2:** Baseline characteristics according to frailty or not stratified by gender

	Female (n = 706)			Male (n = 665)			Interaction *p* value
	Non-frail *n* = 665	Frail *n* = 41	*p* value	Non-Frail *n* = 630	Frail *n* = 35	*p* value	
Age (years)	59.4 (54.8–67.5)	72.4 (65.7–81.3)	< 0.001	62.0 (55.4–71.0)	78.3 (71.2–82.0)	< 0.001	0.633
Smoking (%)	24 (3.4)	2 (4.9)	0.644	214 (34.0)	13 (37.1)	0.716	0.751
Academic year	6 (0–9)	0 (0–5)	< 0.001	6 (6–12)	3 (0–6)	< 0.001	0.762
**Body Composition**							
BMI	23.9 (21.9–26.3)	25.2 (22.0–28.4)	< 0.021	24.5 (22.4–26.5)	23.4 (21.2–25.9)	0.041	0.003
Waist circumference (cm)	80 (74–86)	88 (78.8–95.8)	< 0.001	86 (81–92)	87 (78–97)	0.725	0.007
WHtR	0.52 (0.48–0.56)	0.58 (0.53–0.64)	< 0.001	0.52 (0.49–0.56)	0.54 (0.48–0.59)	0.301	0.001
Total body fat (%)_DXA	37.1 (33.0–41.1)	39.5 (34.3–43.0)	0.128	25 (20.7–29)	23.1 (17.8–31.5)	0.722	0.434
LBM (kg)	34.85 (32.61–37.34)	33.50 (31.80–36.10)	0.025	47.92 (43.91–52.18)	44.66 (40.32–48.01)	< 0.001	0.383
RASM (kg/m^2^)	6.17 (5.76–6.65)	6.17 (5.72–6.71)	0.640	7.87 (7.3–8.4)	7.05 (6.61–7.52)	< 0.001	0.138
Low muscle mass	80 (12.0)	7 (17.1)	0.328	52 (8.3)	7 (20.0)	0.028	0.324
**Underlying disease**							
Metabolic syndrome (%)	172 (25.9)	15 (36.6)	0.145	136 (21.6)	11 (31.4)	0.207	0.989
Hypertension (%)	215 (32.3)	15 (36.6)	0.608	217 (34.4)	18 (51.4)	0.046	0.289
CCI	0 (0–1)	2 (0–2)	< 0.001	0 (0–1)	2 (1–3)	< 0.001	0.074
**Medication**							
Anti-hypertension agents (%)	101 (15.2)	11 (26.8)	0.074	114 (18.1)	8 (22.9)	0.500	0.446
Statin (%)	36 (5.4)	2 (4.9)	1.000	19 (3.0)	2 (5.7)	0.304	0.467
**Laboratory data**							
Total Cholesterol (mg/dl)	205 (182–226)	195 (165.5–213)	0.018	191 (171–213)	176 (168–205)	0.151	0.246
HDL (mg/dl)	58 (50–69)	54 (49.5–63.5)	0.093	50 (43–57)	46 (38–53)	0.033	0.456
LDL (mg/dl)	121 (101–142.5)	114 (90.5–141.5)	0.252	119 (99–139.3)	116 (102–135)	0.568	0.414
Fasting glucose (mg/dl)	93 (88–99)	94 (87–103)	0.470	94 (89–100)	95 (89.5–99)	0.978	0.317
HbA1c (%)	5.8 (5.6–6.0)	5.8 (5.7–6.1)	0.337	5.7 (5.4–5.9)	5.8 (5.5–6.0)	0.355	0.914
Triglyceride (mg/dl)	96 (71–131)	115 (88.5–143)	0.035	107 (77–150)	99 (73–149)	0.574	0.167
UA	5.1 (4.4–5.9)	5.0 (4.4–6.3)	0.578	6.4 (5.5–7.3)	7.2 (5.7–8.6)	0.055	0.367
eGFR (ml/min/1.73m^2^)	94.6 (85.6–100.6)	85.5 (66.7–92.9)	< 0.001	88.4 (77.5–95.6)	74.6 (63.6–82.9)	< 0.001	0.962
**Mortality**	61 (9.2)	9 (22.0)	0.014	60 (9.5)	17 (48.6)	< 0.001	0.031

Values are median (25th–75th percentile) or *n* (%)

*BMI* body mass index: *WHtR* waist-to-height ratio: *BMD* bone mineral density: *LBM* lean body mass: *RASM* relative appendicular skeletal muscle: *CCI* Charlson’s Comorbidity Index: *HDL* high density lipoprotein: *LDL* low density lipoprotein: *HbA1c* hemoglobin A1c: *UA* uric acid: *eGFR* estimated glomerular filtration rate

In examining the individual physical frailty criteria, self-reported exhaustion was significantly more prevalent among women than men in frail participants (Supplement Fig. 1a). Different from the characteristics of frail, pre-frail women were more likely than men to meet the criteria for low activity; otherwise, prefrail men were greater of slowness (Supplement Fig. 1b.).

**Fig. 2:**
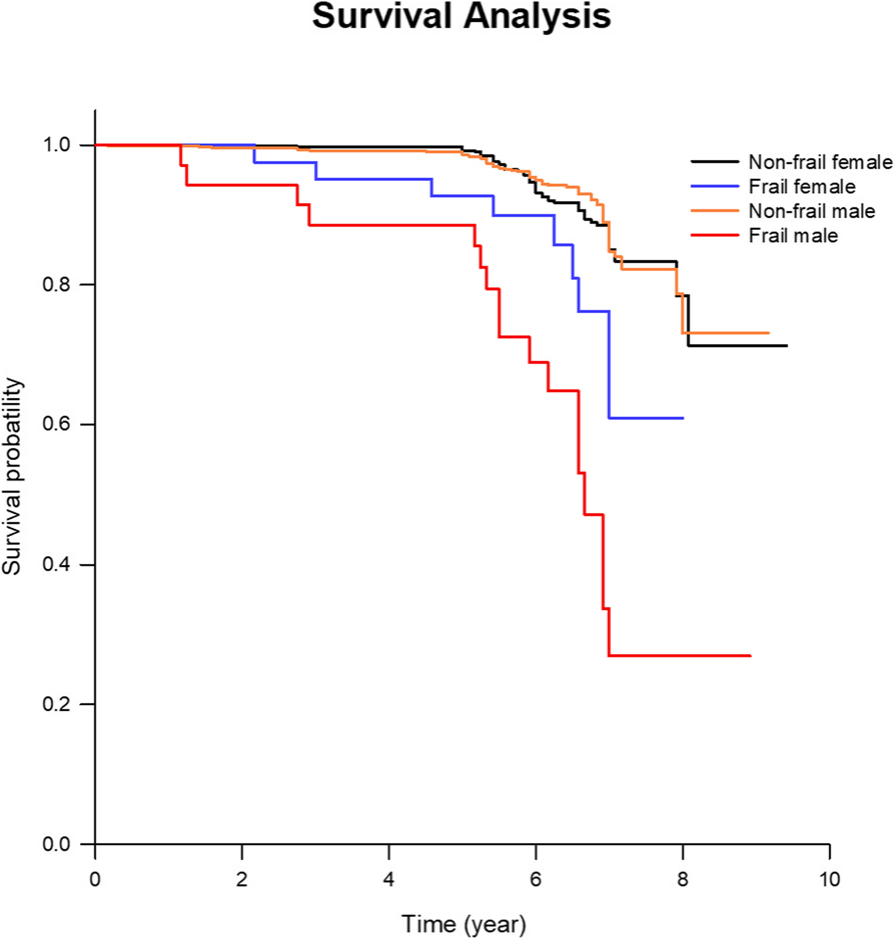
Kaplan-Meier analysis of survival separated by sex and frailty status. Log rank *p* value < 0.001

In the adjusted multivariate analyses, mortality was associated independently with age [HR (95% CI) = 1.038 (1.005–1.073)] and low muscle mass [HR (95% CI) = 2.243 (1.275–3.946)] among women, and with age [HR (95% CI) = 1.060 (1.028–1.092)], smoking [HR (95% CI) = 1.926 (1.190–3.119)] and frailty [HR (95% CI) = 3.395 (1.809–6.371)] among men (Table 3). The survival rate was lowest for frail men (Log ran*p* < 0.001; Fig. 2).

**Table 3:** Cox regression analysis of all-cause mortality separated by sex

	Female (n = 706)		Male (*n* = 665)		Interaction *p* value¶
Variable	Univariate	Multivariate	Univariate	Multivariate	
Age	1.074 (1.047–1.102)^**^	1.034 (1.001–1.068)^*^	1.083 (1.058–1.108)^**^	1.059 (1.028–1.091)^**^	
Gender	–		–	–	
BMI	0.994 (0.930–1.063)		0.881 (0.816–0.953)^#^	0.969 (0.895–1.052)	
Waist circumference	1.013 (0.987–1.039)		0.991 (0.964–1.018)		
CCI	1.476 (1.295–1.683)^**^	1.246 (1.026–1.514)^*^	1.541 (1.300–1.826)^**^	0.888 (0.684–1.153)	
Smoking	1.574 (0.632–3.918)		1.897 (1.213–2.966)^#^	1.975 (1.219–3.203)^#^	
Hypertension	1.870 (1.168–2.994)^#^	1.417 (0.833–2.410)	1.539 (0.981–2.413)	1.272 (0.782–2.071)	
Metabolic syndrome	1.417 (0.871–2.306)	1.189 (0.622–2.272)	0.900 (0.519–1.560)		
Total body fat (%)	1.008 (0.971–1.047)		0.985 (0.951–1.019)		
Low muscle mass	2.433 (1.408–4.206)^#^	2.294 (1.303–4.039)^#^	1.507 (0.796–2.854)		
Total Cholesterol	0.994 (0.987–1.001)	1.012 (0.998–1.026)	0.997 (0.991–1.004)		
HDL	0.993 (0.975–1.010)		1.013 (0.996–1.030)	1.013 (0.995–1.031)	
LDL	0.990 (0.982–0.997)^#^	0.982 (0.967–0.997)^*^	0.997 (0.990–1.003)		
TG	1.003 (0.999–1.007)	1.001 (0.996–1.006)	0.996 (0.992–1.000)^*^	0.997 (0.993–1.001)	
UA	1.119 (0.928–1.350)		0.959 (0.819–1.123)		
FBG	1.008 (0.987–1.030)		1.007 (0.985–1.029)		
eGFR_EPI	0.964 (0.952–0.977)^**^	0.991 (0.972–1.009)	0.975 (0.964–0.987)^**^	0.984 (0.968–1.001)	
Frailty	2.489 (1.235–5.017)^#^	1.400 (0.627–3.125)	7.224 (4.203–12.415)^**^	3.652 (1.926–6.926)^**^	0.031

*BMI* body mass index: *WHtR* waist-to-height ratio: *BMD* bone mineral density: *LBM* lean body mass: *RASM* relative appendicular skeletal muscle: *CCI* Charlson’s Comorbidity Index: *HDL* high density lipoprotein: *LDL* low density lipoprotein: *HbA1c* hemoglobin A1c: *UA* uric acid: *eGFR* estimated glomerular filtration rate

¶Interaction of sex and frailty, adjust variables: age, BMI, CCI, HTN, smoking, frailty, eGFR, TG, LDL, low muscle mass

^*^p value < 0.05

^**^p value < 0.001

^#^*p* value < 0.01

## Discussion

In this study, the prevalence of frailty did not differ between men and women aged > 50 years. The mortality rate was higher for frail than for non-frail subjects. Frail men had significantly less muscle mass and central obesity and were thinner than non-frail men, whereas frail women had a higher BMI, WC and WHtR, but not reduced muscle mass compared with non-frail women. Frailty was an independent risk factor for all-cause mortality for men, but not women. Thus, the main study finding is that the frailty phenotype is a more pronounced indicator of poor health among men than among women.

Our results differ from previous findings in that women was not more prevalent than men, possibly because healthier subjects were enrolled in our study. Although frailty increases the risk of mortality under various assessing approaches, the prevalence of frailty was heterogeneous. The results include that females were more prevalent of frailty by Fried frailty criteria [10], and frailty index [33, 34] and the contrary result even using the same evaluation methods of frailty [15, 35, 36]. Sex may have a different impact on the association of frailty using physical frailty or accumulation deficit model [15, 36]. In this study, we used physical criteria to analyze the relationship between frailty and mortality between sexes; we also incorporated body composition in the analysis of frailty, which was a lack of investigation in the previous studies.

### Frailty in relation to sex differences in body composition and cultural factors

Frailty involves complex interplay among biological, behavioural and social factors, which may underpin the sex difference therein. Our study revealed opposing trends in body composition among frail men and women. Lower muscle mass was more prevalent among frail men than among frail women but was an independent mortality risk factor only for women in analyses adjusted for multiple variables. Our findings demonstrate that frail men reflect more cachexia condition and adequately capture the health deficits than do women.

Consistent with our results, women have been found to be more likely to self-report exhaustion than other clinical frailty components [35]. However, women appear to be more likely to demonstrate difficulty with instrumental activities of daily living, non-lethal functional problems and depressive symptoms [37]. Both men and women report fatigue, but women exhibit more related psychosocial problems, whereas men more often report disability [38]. From a pathophysiological perspective, the chronic disease hypothesis may explain these patterns. Sex differences in multimorbidity have been reported and women presented functional impairment but the non-fatal spectrum of diagnoses [39]. Central obesity, which may have non-fatal sequelae such as osteoarthritis, has more significant impacts and leads to lengthier disability in females than in males [37, 40].

### Limitations

This study has several limitations. First, as it was observational, the adjustment of the analyses may not have covered all potential confounding factors. Second, frail status was not reassessed again in this study; therefore, we could not know the dynamic change of frailty during follow-up duration, like non-frail (including prefrail and robust) became frailty. Third, there are numerous differences between sex and probably related to frailty, including biological factors, nutrition, frequency of exercise and the social aspects are the possible confounders which could not be wholly excluded. Fourth, as disabled subjects and those unable to complete the physical function evaluation were not enrolled in the ILAS, the prevalence of frailty may have been underestimated. Fifth, confined by the cohort longitudinal observational study, the possible underlying pathophysiology could not be elucidated. The intervention to reverse frailty and the generalisability of our findings must be investigated in further research.

## Conclusion

Although the prevalence of frailty did not differ between sexes in this study, the impact of frailty on all-cause mortality was more pronounced for men than for women. The observed sex disparities in the effects of frailty on health outcomes warrant further exploration.

### Supplementary Information

**Supplementary Material 1.**

**Supplementary Material 2.**

## Data Availability

The datasets analysed during the current study available from the corresponding author on reasonable request. Please contact the corresponding author, Po-Hsun, Huang (huangbsvgh@gmail.com ), for data access.
