# Supplementary material for: Sex differences in the frailty phenotype and mortality in the I-Lan longitudinal aging study cohort
Source: BMC Geriatr. 2024 Feb 23;24:182. doi: 10.1186/s12877-024-04785-w (PMC10893742; doi:10.1186/s12877-024-04785-w)
Supplement: Supplementary file 2 — Supplementary Material 2. [file 12877_2024_4785_MOESM2_ESM.docx]

**Supplement Figure 1. Percentage of Frailty Components separated by sex in frail and prefrail participants**

1. **Frail**


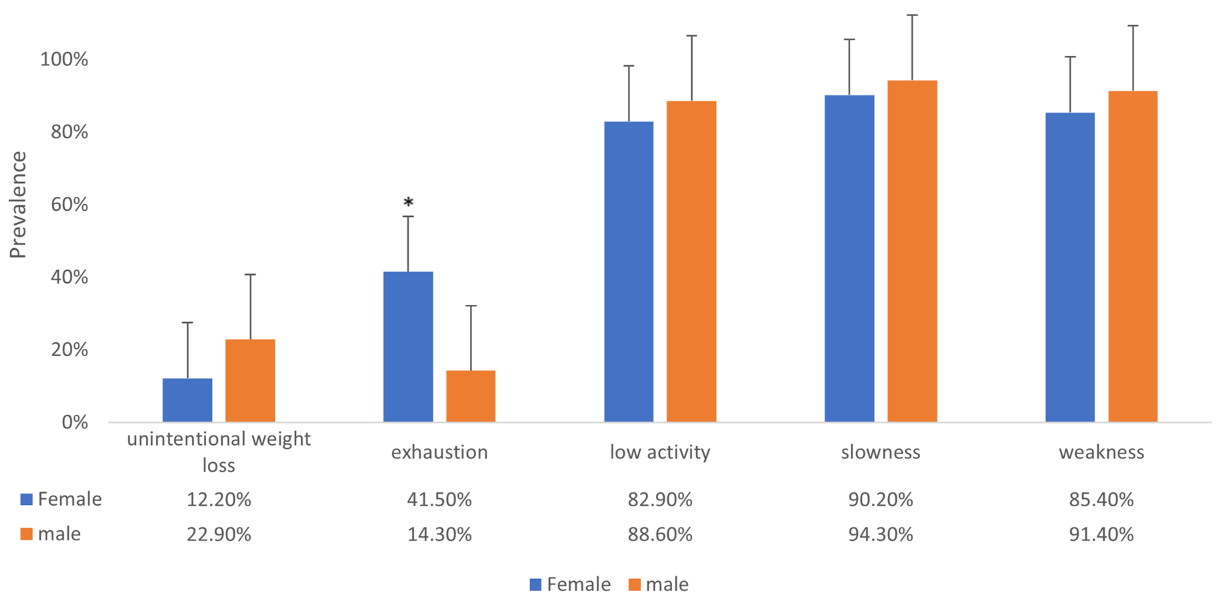


* *p value* < 0.05

1. **Pre-frail**

**
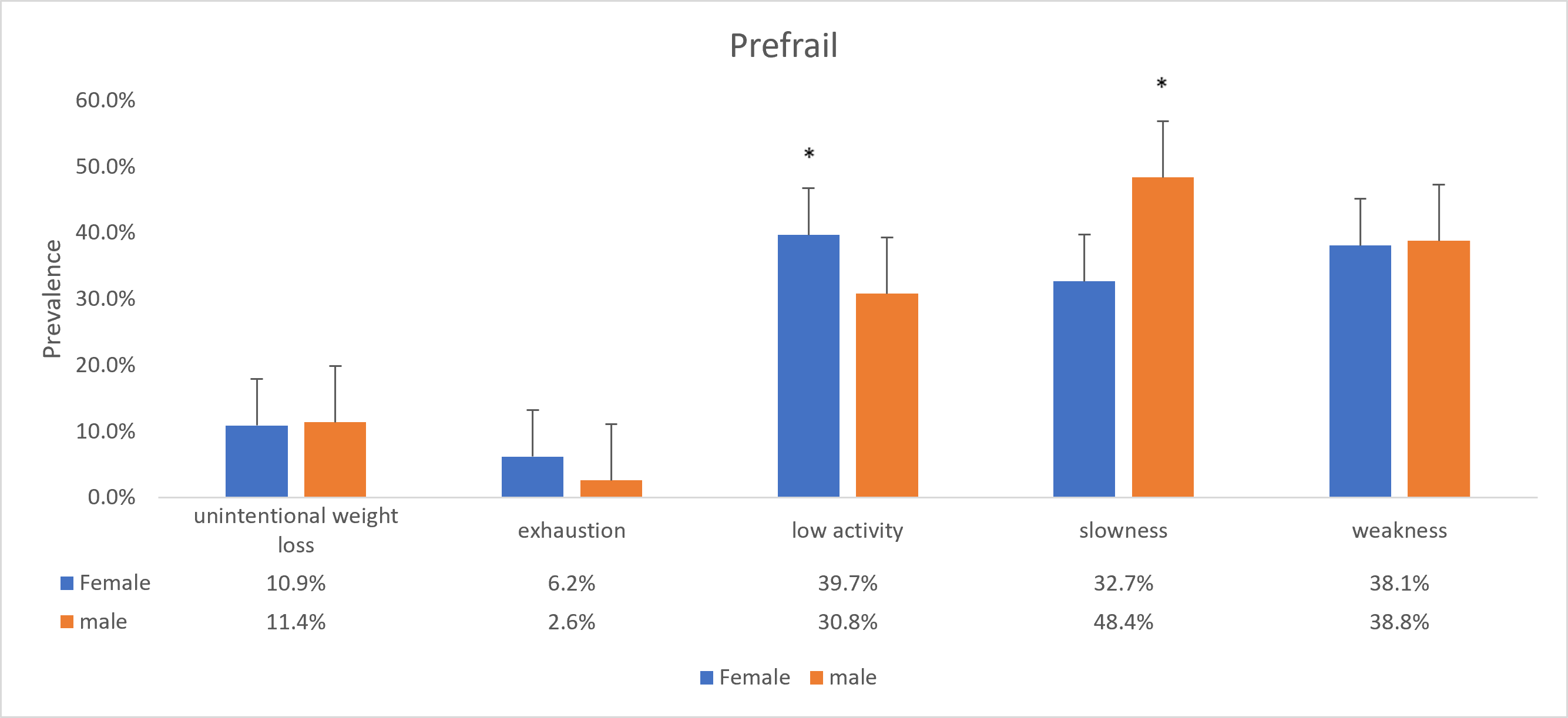
**

* *p value* < 0.05
